# Identification of two novel lipid metabolism-related long non-coding RNAs (SNHG17 and LINC00837) as potential signatures for osteosarcoma prognosis and precise treatment

**DOI:** 10.1186/s12920-023-01553-4

**Published:** 2023-05-25

**Authors:** Zhifang Tang, Hanzhen Feng, Longjun Shu, Minzheng Guo, Baochuang Qi, Luqiao Pu, Hongxin Shi, Junxiao Ren, Chuan Li

**Affiliations:** 1 Clinical Medical College of Dali University, Dali, Yunnan, 671000 China; 2 Department of Orthopedics, The First People’s Hospital of Dali City, Yunnan, 671000 Dali China; 3 Department of Orthopedics, Kunming Medical University, Kunming, Yunnan China; 4 Department of Orthopedics, The 920th Hospital of Joint Logistics Support Force of Chinese People’s Liberation Army, Kunming, Yunnan China; 5 Department of Orthopedics, Yunnan University of Chinese Medicine, Kunming, Yunnan China

**Keywords:** Long non-coding RNA, Osteosarcoma, Lipid metabolism, Bioinformatics analysis, Competing endogenous RNA networks

## Abstract

**Objective:**

Dysregulated lipid metabolism enhances the development and advancement of many cancers, including osteosarcoma (OS); however, the underlying mechanisms are still largely unknown. Therefore, this investigation aimed to elucidate novel potential lipid metabolism-related long non-coding RNAs (lncRNAs) that regulate OS development and provide novel signatures for its prognosis and precise treatment.

**Materials and methods:**

The GEO datasets (GSE12865 and GSE16091) were downloaded and analyzed using R software packages. Immunohistochemistry (IHC) was used to evaluate protein levels in OS tissues while real-time qPCR was used to measure lncRNA levels, and MTT assays were used to assess OS cell viability.

**Results:**

Two lipid metabolism-associated lncRNAs (LM-lncRNAs), small nucleolar RNA host gene 17 (SNHG17) and LINC00837, were identified as efficient and independent prognostic indicators for OS. In addition, further experiments confirmed that SNHG17 and LINC00837 were significantly elevated in OS tissues and cells than para-cancerous counterparts. Knockdown of SNHG17 and LINC00837 synergistically suppressed the viability of OS cells, whereas overexpression of the two lncRNAs promoted OS cell proliferation. Moreover, bioinformatics analysis was conducted to construct six novel SNHG17-microRNA-mRNA competing endogenous RNA (ceRNA) networks, and three lipid metabolism-associated genes (MIF, VDAC2, and CSNK2A2) were found to be abnormally upregulated in OS tissues, suggesting that they were potential effector genes of SNHG17.

**Conclusion:**

In summary, SNHG17 and LINC00837 were found to promote OS cell malignancy, suggesting their use as ideal biomarkers for OS prognosis and treatment.

**Supplementary Information:**

The online version contains supplementary material available at 10.1186/s12920-023-01553-4.

## Introduction

Osteosarcoma (OS) is a primary malignancy associated with mesenchymal tissue that occurs mainly in adolescents and children [1]. The treatment of OS has not changed significantly in the past five decades. About 35–45% of OS patients are chemotherapy-resistant, show low treatment responses, and are prone to distant metastasis. The 5-year survival rate for OS individuals with distant metastases is < 20% [2]. Although the identification of effective treatment strategies is the focus of current research, assessment systems, and reliable disease predictors are also essential for clinical practice to determine the treatment response, monitor disease recurrence and metastasis, support clinical decisions, and predict survival outcomes.

Many recent studies have suggested that the metabolic reprogramming of cells plays a crucial part in tumor development and progression [3]. Metabolic reprogramming is a process by which tumor cells regulate their synthesis and catabolism to obtain the required energy and substances to survive extreme microenvironments, and involves carbohydrates, lipids, amino acids, and nucleotides [4]. Of these, aberrant fatty acid metabolism, in particular, has been intensively investigated [5]. Modulation of lipid metabolism, including lipid uptake, synthesis, and hydrolysis, is crucial for maintaining cellular homeostasis. Nutrients are required by the developing tumor to sustain its proliferation, survival, and metastatic growth, and these are obtained from the tumor microenvironment [6]. The literature indicates that genes associated with lipid metabolism are prognostic indices for diffuse glioma, renal and hepatocellular carcinomas, and ovarian cancer [7–10]. Unfortunately, lipid metabolism-associated genes involved in the initiation, growth, and prognosis of OS prognosis are poorly understood.

Long non-coding RNAs (lncRNAs) contain > 200 nucleotides and lack protein-coding ability. The intracellular abundance of lncRNAs is between 70% and 98%, with a length range of 200 nt to 100 kb [11]. Although lncRNAs do not encode protein, their expression is critical during tissue development and in multiple regulatory pathways, including chromatin modification, intranuclear transport, X chromosome silencing, genomic imprinting, and transcriptional interference and activation [12–14]. In OS, abnormal lncRNA levels are associated with patient survival times, where the correlations are mostly negative. Recent research indicates that lncRNAs could function as effective tumor biomarkers due to their sensitivity, noninvasive properties, and specificity [15]. However, the involvement of lipid metabolism-associated lncRNAs (LM-lncRNAs) in OS prognosis is still undetermined.

The present investigation used a variety of bioinformatics methods to comprehensively elucidate LM-lncRNAs and use these to establish a novel risk score (RS) model to evaluate their prognostic importance in OS. Furthermore, the modulatory activity of LINC00837 and SNHG17 (lncRNAs) on the expression of downstream gene targets was verified in tissue samples from OS patients. The data of this investigation may provide theoretical insights for better prognosis and subsequent OS treatment.

## Materials and methods

### Data acquisition

The gene expression dataset (GSE12865 [16]) was acquired from Gene Expression Omnibus (GEO, https://www.ncbi.nlm.nih.gov/Geo/) of the National Center for Biotechnology Information (NCBI). GSE12865 included 12 pediatric OS samples and 2 healthy osteoblast cell samples from the GPL6244 platform (Affymetrix Human Gene 1.0 ST Array). RNA-sequencing (RNA-seq) data from 88 OS patients were obtained from TCGA (https://portal.gdc.cancer.gov). Recent clinical information from OS individuals was obtained from the TARGET (https://ocg.cancer.gov/programs/target) database. Lastly, the clinical and RNA-seq data of 85 OS patients were used as the training cohort (TCGA-TARGET-OS), with the validation cohort (GSE16091 [17]) comprising data from 34 OS patients with corresponding features to the GSE16091 dataset. The inclusion criteria for the GEO datasets were: (1) Inclusion of transcriptomic data including LM-lncRNAs; (2) Availability of basic information on OS aggressiveness and the tumor microenvironment; (3) Samples sized of greater than 20.

### Determination of lipid metabolism-associated differentially expressed genes (DEGs)

Twenty-one lipid metabolism-associated pathways and five gene sets associated with lipid metabolism were identified from the Kyoto Encyclopedia of Genes and Genomes (KEGG) (http://www.kegg.jp/blastkoala/) and the Molecular Signatures Database (MisDB) (https://www.gseamsigdb.org/gsea/msigdb/index.jsp), respectively (Supplementary Table S1, reference: Lipid metabolic gene-wide profile and survival signature of lung adenocarcinoma). The keywords used for the search included sterol lipids, prenol lipids, polyketides glycerophospholipids, glycerolipids, sphingolipids, fatty acyls, and saccharolipids. After the removal of overlapping genes, 1045 genes associated with lipid metabolism were identified. The R package “limma” (Version R-4.2.3) was used to identify DEGs associated with lipid metabolism in 12 pediatric OS and 2 healthy osteoblast cell samples from the GSE12865 dataset. The criteria for DEG identification were |log2 fold change| (logFC) > 1 and false discovery rate (FDR) < 0.05.

### Construction and evaluation of risk score

First, the most abundant lncRNAs were identified in the GSE16091 dataset and TCGA data. Pearson correlation and univariate Cox regression (UCRA) analyses were then conducted to identify LM-lncRNAs with cor > 0.3 and P < 0.05, respectively, and to identify lncRNAs significantly linked with survival. This led to the identification of four LM-lncRNA which were then used to construct prognostic signatures with multivariate Cox regression analysis (MCRA). Lastly, the RS was calculated as ExpGene1 × Coef 1 + ExpGene2 × Coef 2 + ExpGene3 × Coef 3, where Coef = coefficient and Exp = each signature gene’s normalized expression value. The RS system of the 2 LM-lncRNA was established in the training cohort and confirmed in the validation cohort. The participants were grouped into high- and low-risk groups on the basis of the median RS. UCRA and MCRA were then conducted to determine if the RS was an independent prognostic factor for the survival of OS patients.

### Correlation of the risk score with immune cell infiltration in OS

Single-sample gene set enrichment analysis (ssGSEA) was carried out to determine the enrichment scores of 24 immune cell types in each sample. The immune cells included effector memory CD8-T, type-17 T-helper, natural killer (NK), memory B, central memory CD4-T, immature dendritic, activated CD4-T, MDSC neutrophil, regulatory T, mast, plasmacytoid dendritic, activated B, central memory CD8-T, type-1 T-helper, eosinophil, activated CD8-T, type-2 T-helper, effector memory CD4-T, immature CD56bright natural killer, B, macrophage, T follicular helper, activated dendritic, CD56dim NK, gamma delta T, monocyte, and NK-T cells. The association of RS with immune cell infiltration was evaluated using Spearman correlations.

### Nomogram establishment

MCRA was used to construct a nomogram for overall survival and calibration curves to assess the prediction capability of the nomogram. Decision curves were drawn to assess the nomograms’ clinical application by measuring the net benefits at the threshold probabilities range.

### Constructing the competing endogenous RNA (ceRNA) network

First, the correlations between LINC00837 and SNHG17 with the expression of various lipid metabolism-associated genes were assessed by Cytoscape v3.9.0. Target miRNAs were predicted using the StarBase database, and their corresponding target mRNAs were selected via the miRDIP v4.1 e-tool (http://ophid.utoronto.ca/mirDIP/index_confirm.jsp). Lastly, Cytoscape v3.9.0 was used to construct a ceRNA network incorporating the regulatory relationships between lncRNA-miRNA and miRNA-mRNA.

### Functional enrichment analysis

Gene set enrichment analysis (GSEA) was carried out to identify the most enriched pathways in the high- and low-risk cohorts, and Gene Ontology (GO) annotations and KEGG pathway assessments were carried out for lipid metabolism-related DEGs (|log2FC|≥ 1, FDR < 0.05) associated with SNHG17 and LINC00837 using the “clusterprofiler” R package (Version R-4.2.3).

### Clinical samples collection and immunohistochemistry (IHC)

Primary OS tumor and healthy tissues were provided by the 920th Hospital of Joint Logistics Support Force of the Chinese People’s Liberation Army, Department of Orthopedics. Protein levels of MIF, CSNK2A2, VDAC2, and ODC1 in both tumor and para-cancerous tissues, were evaluated by IHC. Briefly, the tissues were thawed at room temperature and were subsequently boiled in sodium citrate at 80–90℃ for 25 min and incubated with sodium citrate containing 0.1% H_2_O_2_ in Tris-HCl for 1.5 h for rehydration and antigen retrieval. Tissue sections were then incubated with 3% H_2_O_2_ solution for 10 min, followed by incubation with 5% bovine serum albumin (FBS, Sigma, USA). Next, the samples were incubated with primary antibodies against MIF (1:500, Abcam, UK), CSNK2A2 (1:500, Abcam), VDAC2 (1:500, Abcam), and ODC1 (1:500, Abcam) at 4 °C overnight. The samples were then washed three times with Tris washing buffer and probed with biotinylated secondary antibodies (Abcam) and stained according to the instructions provided by the manufacturer of the commercial IHC kit (ThermoFisher Scientific, USA), followed by counterstaining and visualization with the ChemMate DAKO EnVision Detection kit (DAKO) and evaluation under light microscopy (ThermoFisher Scientific) by two experienced pathologists.

### Cell culture and vector transfection

The human OS cell lines Saos2 and HOS and the normal osteoblast cell line hFOB 1.19 were obtained from the American Type Culture Collection (ATCC, MA, USA). The cells were grown in RPMI-1640 medium (HyClone, UT, USA) supplemented with antibiotics (100 U/mL penicillin and 100 µg/ml streptomycin) and 10% fetal bovine serum (FBS, Gibco, USA) in an incubator with 5% CO_2_ at 37 ℃. The cells were washed with phosphate-buffered saline (PBS, Beyotime, China) for passage and experimental purposes, and the cells were routinely assessed for the presence of mycoplasma. For vector transfection, overexpression and silencing vectors for SNHG17 and LINC00837 were designed and prepared by Sangon Biotech (Shanghai, China). Briefly, the cDNA for the above two genes was obtained and cloned into commercial overexpression plasmids, and the short hairpin RNAs (shRNAs) for SNHG17 and LINC00837 were synthesized. OS cells were pre-cultured in 6-well plates overnight, and the vectors were delivered intracellularly via using the Lipofectamine 2000 reagent (Invitrogen, USA) according to manufacturer’s protocols. The transfection efficiency was evaluated by RT-qPCR.

### Cell viability assay

Cells (1,000 cells per well) in 200 µl of culture medium were grown in 96-well plates for 12 h before the addition of MTT solution (20 µl per well) and incubation for 4 h at 37 ℃. After discarding the culture supernatants, 150 µl per well of DMSO was added to dilute formazan. The plates were vortexed and the optical density (OD) values were measured using a microplate reader (ThermoFisher Scientific) to represent relative OS cell viability.

### Statistical analysis

Differences in overall survival between the two groups were assessed by Kaplan-Meier curves and log-rank tests, and the predictive accuracy of the risk model was evaluated using receiver operating characteristic (ROC) curves and area under the curve (AUC) values. Student’s t-tests were conducted for inter-subgroup comparison of the RS data based on the following clinicopathological variables: age = 16 years, sex = both, and metastatic status. All data were analyzed using R software and P-values < 00.05 were considered statistically significant.

## Results

### Identification of lipid metabolism-associated genes and lncRNAs in OS patients

**Fig. 1:**
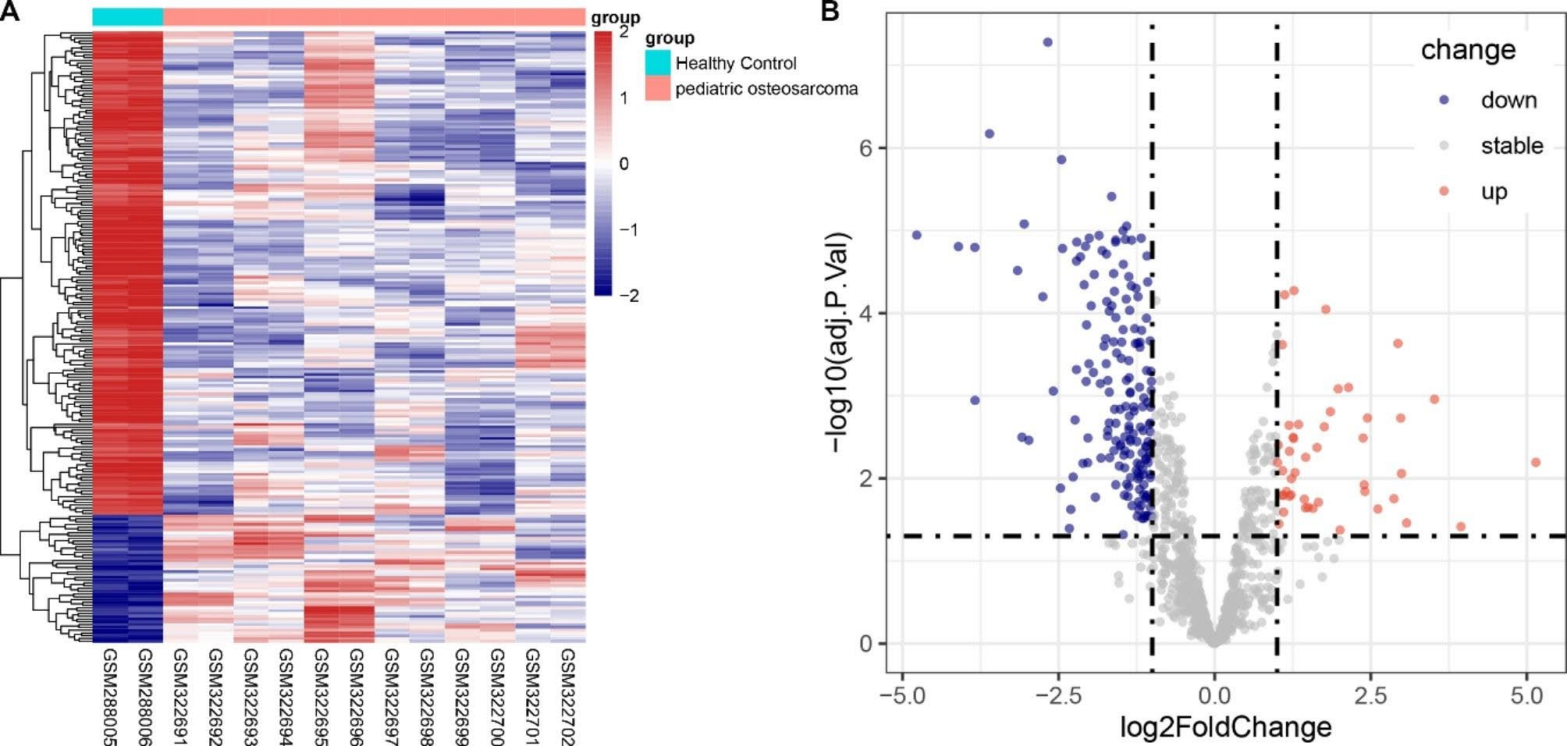
Analysis of differentially expressed genes. A: Heatmap showing hierarchical clustering of the DEGs. B: Volcano plot showing the DEGs between OS patients and healthy controls

A total of 219 DEGs associated with lipid metabolism were identified in the GSE12865 dataset (Supplementary Table S2). These are shown by a volcano plot (Fig. 1B), and to represent their hierarchical clustering analysis, a heatmap (Fig. 1A). After the removal of the lncRNA data from training and validation cohorts, 116 common lncRNAs were identified. Pearson correlation coefficients were used to determine the associations between these 116 common lncRNAs and the DEGs, resulting in the identification of 113 LM-lncRNAs (Supplementary Table S3) which were used for further investigations.

### Identification of prognostic lipid metabolism-related lncRNAs and risk model construction

**Fig. 2:**
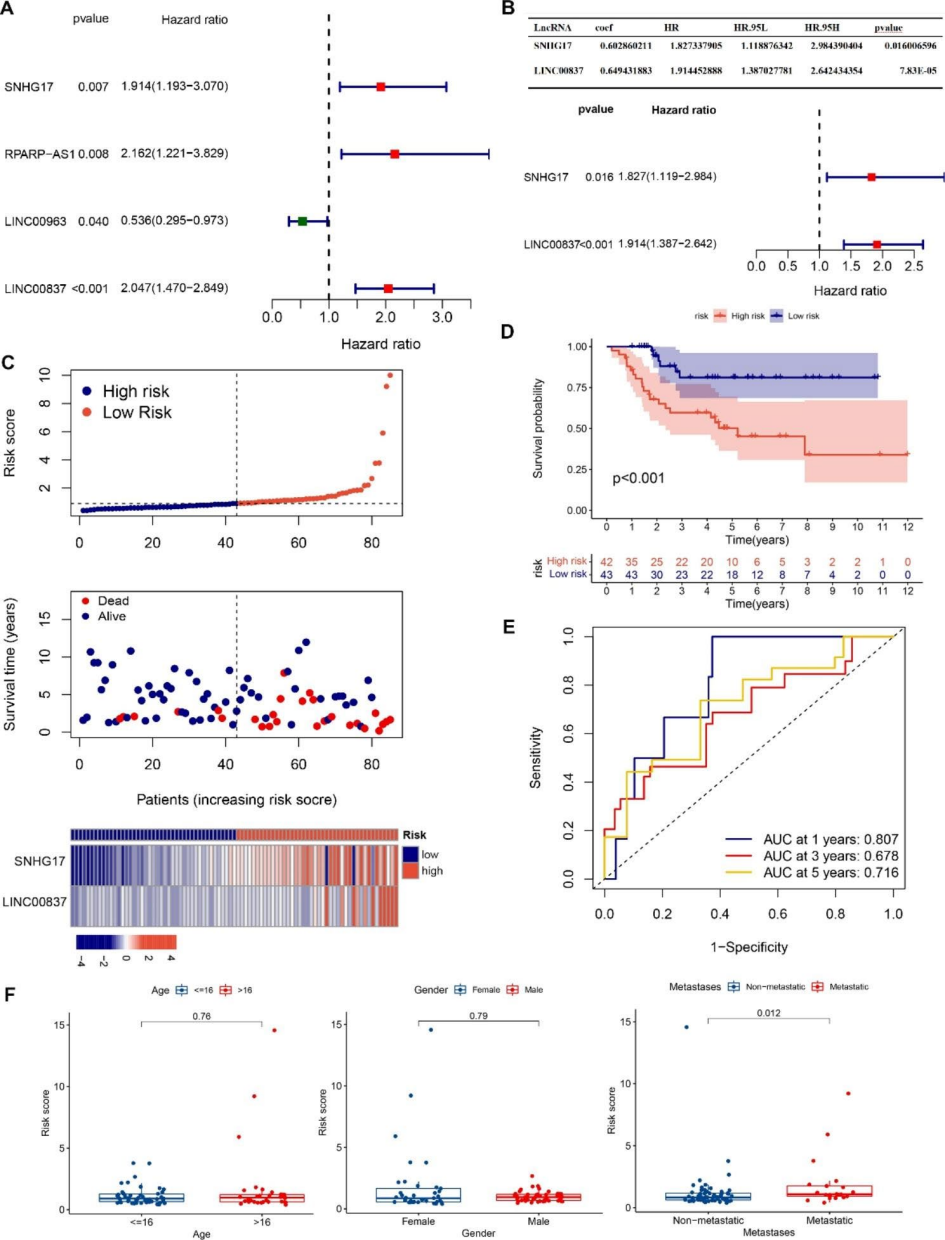
Identification of prognostic markers and risk model construction. A: UCRA forest plot of prognosis-associated lncRNAs. B: MCRA forest plot of prognosis-associated lncRNAs. C: Distribution of survival status, RS, and SNHG17 and LINC00837 expression in the TARGET-OS training cohort. D: Kaplan-Meier curves for the high- and low-risk TARGET-OS groups. E: ROC curves predicting the one-, three-, and five-year overall survival in the TARGET-OS training cohort. F: Correlation of RS with clinical variables (age, metastasis, and sex)

The UCRA of GSE16091 and TARGET-OS further investigated the ability of the LM-lncRNAs to predict OS prognosis, finding that four 4 LM-lncRNAs were significantly associated with the overall survival of OS patients (P < 0.05) (Fig. 2A). Additionally, MCRA was conducted to verify the predictive robustness of the selected LM-lncRNAs in the training cohort. SNHG17 and LINC00837 were used for the construction of the risk model, calculating the RS as 0.60286 × SNHG17 expression value + 0.64943 × LINC00837 expression value (Fig. 2B). The subjects in the training cohort were grouped into high- and low-risk groups according to the median RS. The survival status of OS patients in the training cohort, RS distributions, and the LINC00837 and SNHG17 expression values are shown in Fig. 2C. Kaplan-Meier survival analysis showed that the high-risk group had the worst clinical outcomes (Fig. 2D), and ROC curves indicated significant accuracy of the pooled LM-lncRNA signatures in the training cohort in the prediction of overall survival (AUC = > 0.65) (Fig. 2E). The evaluation of the RS results and clinical and pathological variables of OS patients in the training cohort showed no significant differences in the RS values of patient age ( < = 16 and > 16) and sex (both), whereas patients with metastatic disease had higher RS values than those without metastases (Fig. 2F).

### Validation of SNHG17 and LINC00837 as ideal prognostic biomarkers in the cohort from the GSE16091 dataset

**Fig. 3:**
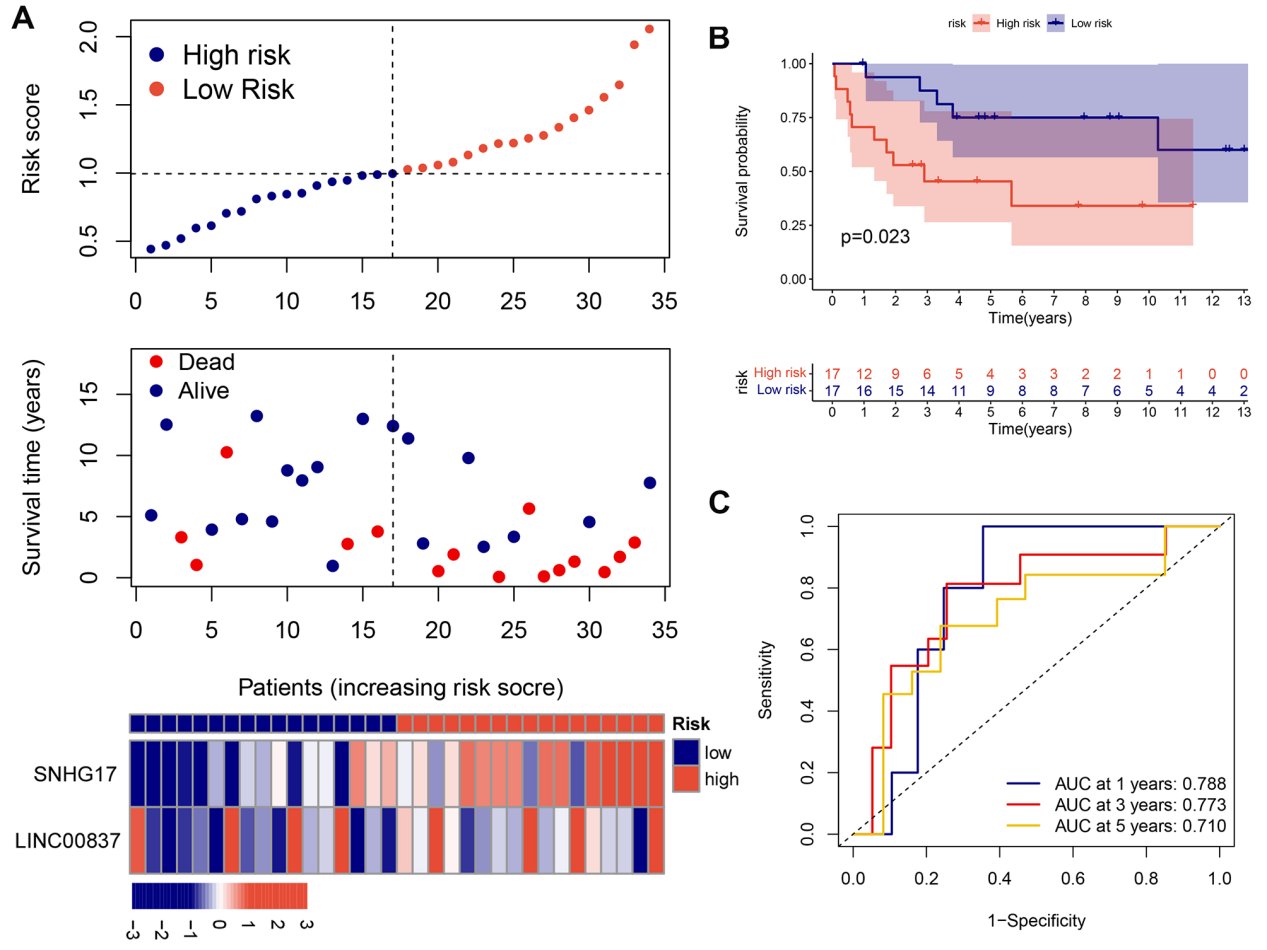
Validation of the risk model. A: Distribution of survival status, RS, and SNHG17 and LINC00837 expression in the GSE16091 training cohort. B: Kaplan-Meier curves for the the high- and low-risk groups in the GSE16091 training cohort. C: ROC curves for the prediction of one-, three-, and five-year overall survival in the GSE16091 training cohort

**Fig. 4:**
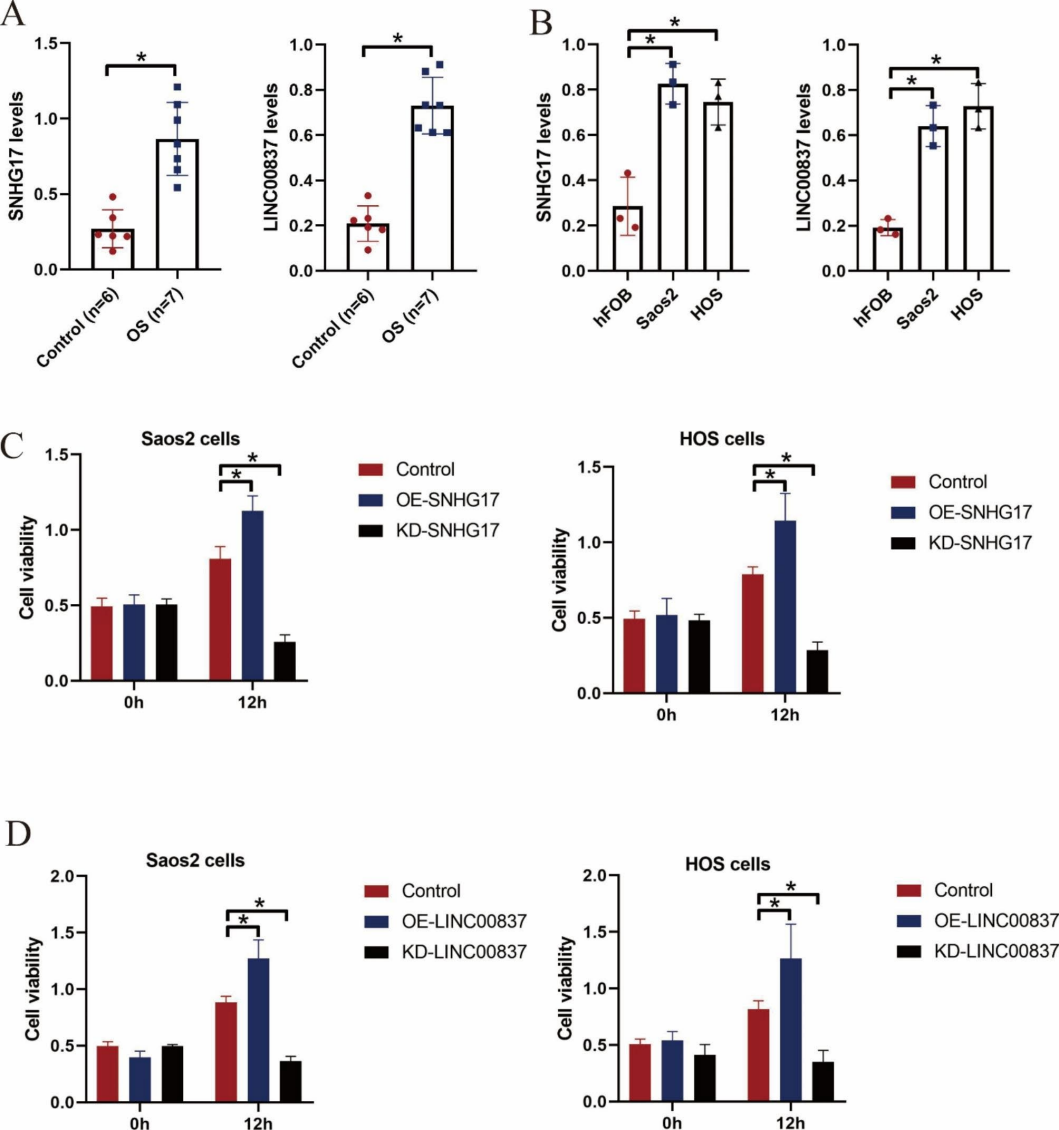
LncRNA SNHG17 and LINC00837 regulate cell viability in OS cells. The levels of SNHG17 and LINC00837 in (A) OS tissues and (B) cell lines were measured by RT-qPCR. (C, D) MTT assays measuring the viability of OS cells. The results are from three independent experiments with *P < 0.05 considered statistically significant

To further verify the risk model in the GSE16091 training cohort, the RS values of the two risk groups were assessed using the previously established formula. This showed that patients with elevated RS values had poorer survival outcomes (Fig. 3A) compared with patients with lower RS values (Fig. 3B). As seen in the heatmap, the levels of LINC00837 and SNHG17 were elevated in the high-risk group (Fig. 3A). Moreover, the ROC curve also indicated the effectiveness of the model in prognosis prediction in the validation cohort comprising the GSE39055 dataset (AUC > 0.7, Fig. 3C). Furthermore, RT-qPCR analysis indicated the abnormal overexpression of both SNHG17 and LINC00837 in OS tissues (Fig. 4A) and cells (Fig. 4B), in contrast to their normal counterparts. MTT assays also showed that silencing of SNHG17 and LINC00837 reduced the viability of OS cells in a time-dependent manner, and overexpression of the two lncRNAs increased OS cell proliferation (Fig. 4C, D), suggesting that SNHG17 and LINC00837 influenced OS cell malignancy.

### SNHG17- and LINC00837-based risk models in OS were related to cell proliferation, mitosis, and immune cell infiltration

To investigate the downstream signaling pathways involved in OS development in the established risk model, GSEA and KEGG pathway assessments were conducted. As shown in Supplementary Figure S1A, the DEGs in the high-risk group were significantly linked with pathways associated with the cell cycle and meiosis, while genes in the low-risk group were significantly associated with cellular functions, including asthma, complement, coagulation disease, and ECM-receptor interactions (Supplementary Figure S1B). Hallmark enrichment demonstrated that the DEGs in the high-risk group were enriched in DNA repair, adipogenesis, and E2F-associated targets (Supplementary Figure S1C), and those in the low-risk group were associated with angiogenesis and API-CAL surface (Supplementary Figure S1D). These results suggested that the selected LM-lncRNAs (SNHG17 and LINC00837) might affect cell proliferation and mitosis-associated signal pathways to regulate OS development. Furthermore, immune cell infiltration is significantly linked with OS prognosis and immune cell functions can be influenced by lncRNAs. The correlations between the infiltration of immune cells in the risk groups were then analyzed (Supplementary Table S4). Spearman correlation analysis indicated that aberrantly expressed LINC00837 and SNHG17 in the risk model were associated with differences in the infiltration of central memory CD8 T cells (P < 0.001, r = -0.32), plasmacytoid dendritic cells (P = 0.03, r = -0.20), natural killer cells (P < 0.001, r = -0.30), and macrophages (P < 0.001, r = -0.25) (Supplementary Figure S2), suggesting that SNHG17 and LINC00837 influenced the infiltration of immune cells during OS development.

### Identification of SNHG17 and LINC00837-based risk models and metastasis as independent OS prognostic factors

**Fig. 5:**
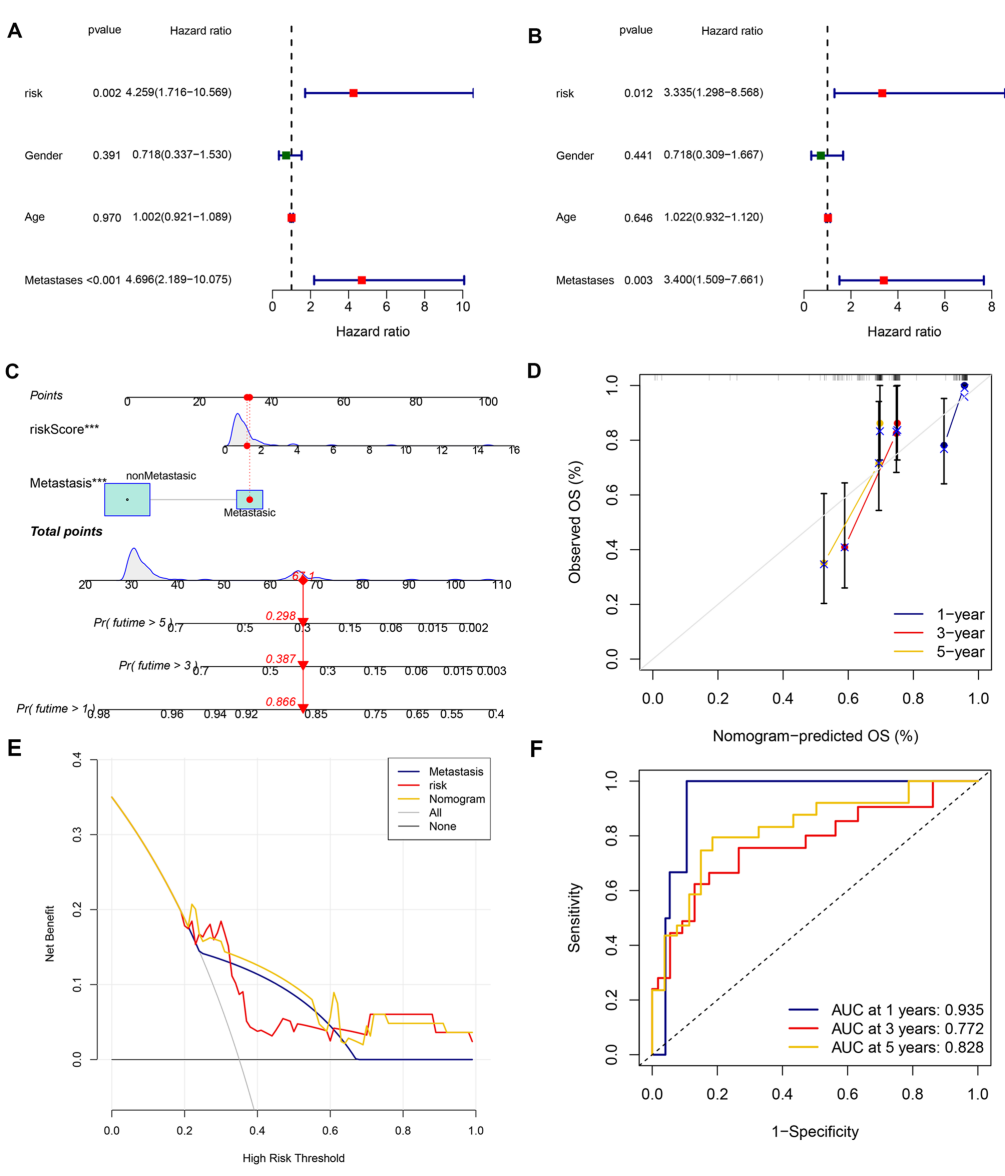
Clinical association of risk score with nomogram development. A: UCRA forest plot of prognosis-associated factors. B: MCRA forest plot of prognosis-associated factors. C: Nomogram for predicting the one-, three-, and five-year overall survival of OS patients. D: Calibration of the nomogram for one-, three-, and five-year overall survival of OS patients; E: Decision curve for the prediction of three-year survival. F: ROC curves for the prediction of one-, three-, and five-year overall survival of OS patients

The UCRA and MCRA analyzed the associations between the clinical variables, SNHG17 and LINC00837-based risk models, age and sex of the patients, and metastasis status with the survival prognosis of OS patients. The UCRA (Fig. 5A) indicated that RS values and metastasis status were closely linked with the prognosis of OS patients. In contrast, age and sex had no effect, consistent with the MCRA data that RS and metastasis were independent predictors of OS prognosis (Fig. 5B). Furthermore, a nomogram based on RS and metastasis predicted the one- three-, and five-year survival of OS patients (Fig. 5C); the findings were consistent with the calibration plots on the one- and three-year overall survival (Fig. 5D). Decision curves were used to determine the magnitude of the correlations, confirming the results (Fig. 5E). The AUC values at one (0.935), three (0.772), and five (0.828) years indicated that the nomograms were effective in predicting survival outcomes (Fig. 5F). The data thus indicated that the constructed risk model and metastasis were independent predictors of survival in OS patients.

### Analysis and construction of SNHG17/LINC00837-microRNA-mRNA competing endogenous RNA networks

**Fig. 6:**
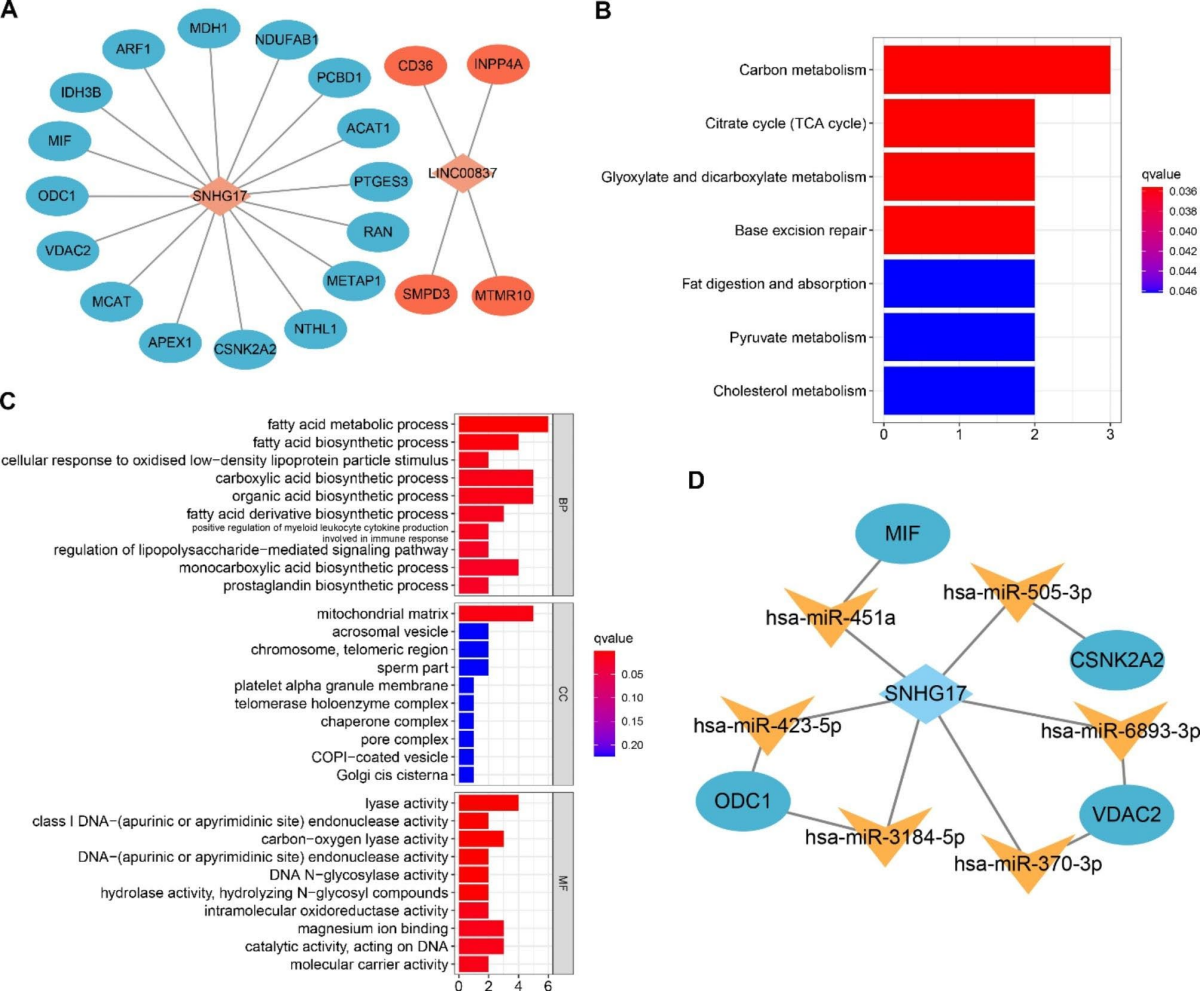
Construction of the ceRNA network. A: Lipid metabolism-linked lncRNA-mRNA network of SNHG17 and LINC00837. B: KEGG enrichment analysis. C: GO annotation analysis. D: The SNHG17/miRNA/lipid metabolism-related gene network in OS patients

Analysis of ceRNA networks has shown that lncRNAs often exert their biological functions by acting as RNA sponges for microRNAs, influencing the stability and degradation of the downstream mRNA [18, 19]. First, the correlations between SNHG17 and LINC00837 and downstream lipid metabolism-associated genes were analyzed, showing that the latter were significantly influenced by both SNHG17 and LINC00837 (Supplementary Table S3). Next, Cytoscape 3.9.0 software was used to construct the lncRNA-mRNA networks, which showed that 16 genes potentially interacted with SNHG17, and 4 genes interacted with LINC00837 (Fig. 6A). KEGG pathway analysis indicated that the selected genes were significantly associated with various metabolic pathways, including those related to carbon, glyoxylic esters, dicarboxylic esters, and the citric acid cycle (Fig. 6B). Furthermore, GO annotation analysis indicated that many of these genes were significantly associated with lipid metabolism (Fig. 6C). Potential microRNAs were screened in the online ENCORI database to assess the combined associations between the microRNAs, SNHG17, LINC00837, and lipid metabolism-related genes to construct lncRNA-microRNA-mRNA ceRNA networks. Interestingly, six SNHG17-miRNA-lipid metabolic gene networks were established, including the SNHG17-miR-451a-MIF axis, SNHG17-miR-423-5p-ODC1 axis, SNHG17-miR-3184-5p-ODC1 axis, SNHG17-miR-370-3p-VDAC2 axis, SNHG17-miR-6893-3p-VDAC2 axis, and SNHG17-miR-505-3p-CSNK2A2 axis (Fig. 6D). Unfortunately, no microRNAs were found to link LINC00837 with CD36, INPP4A, SMPD3, and MTMR10, suggesting that LINC00837 might regulate those genes in a ceRNA-independent manner.

### Examination of the SNHG17-related downstream mRNA targets in OS tissues

**Fig. 7:**
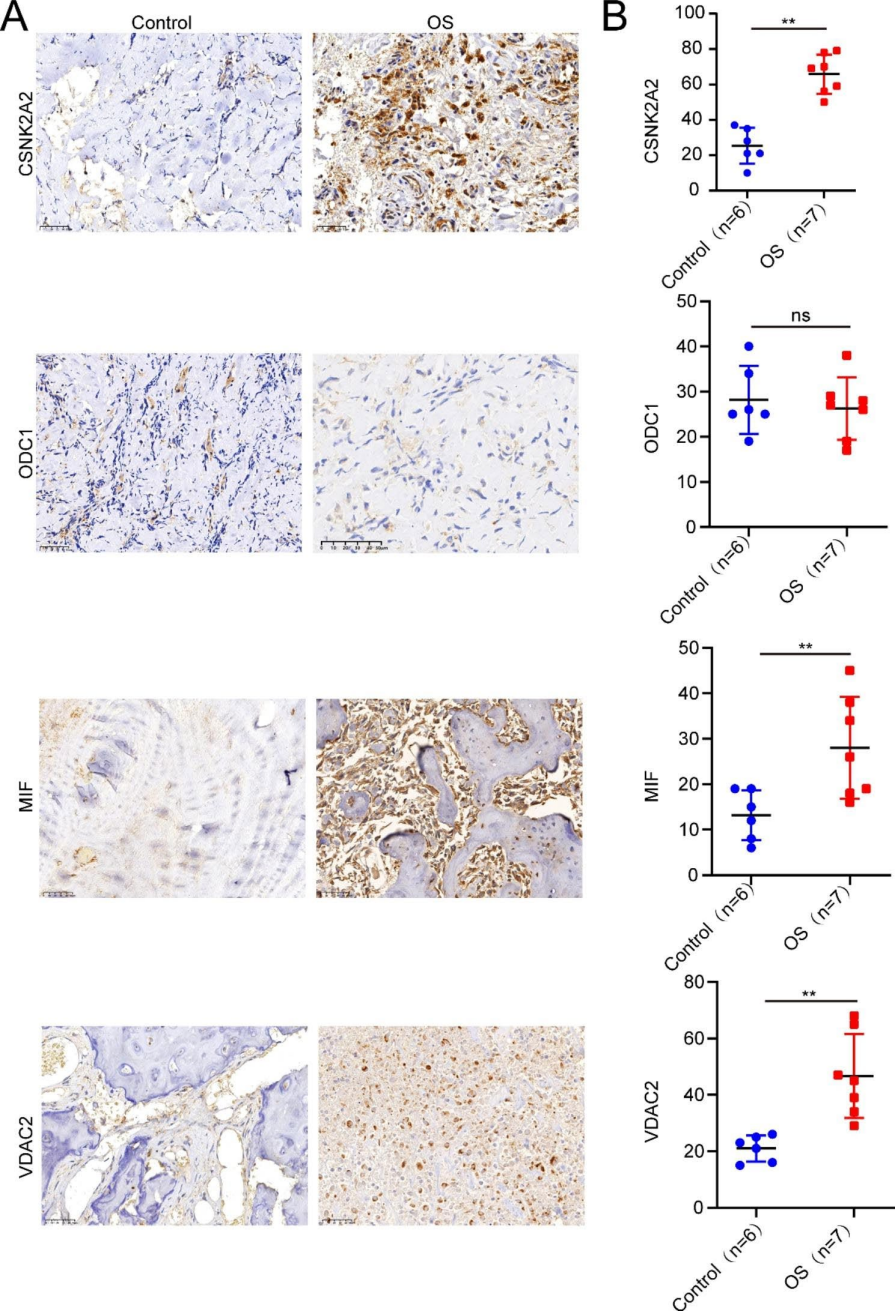
Verification of SNHG17-associated mRNA expression in OS patients. A: Immunohistochemical staining of CSNK2A2, MIF, ODC1, and VDAC2 in tumor and healthy tissues from OS patients. B: Quantification of the expression of CSNK2A2, ODC1, MIF, and VDAC2 in tumor and healthy tissues from OS patients, shown by Student’s t-test. Bars, mean ± SD. *P < 0.05, **P < 0.01, ***P < 0.001; ns, non-significant

Following the establishment of the SNHG17-microRNA-mRNA ceRNA networks and the identification of potential effector genes (CSNK2A2, ODC1, MIF, and VDAC) in SNHG17-mediated biological functions, IHC was performed to examine the expression of these four genes in clinical specimens. Briefly, the tumor (N = 7) and adjacent (N = 6) tissues of OS patients were collected and stained using IHC, showing that CSNK2A2, VDAC2, and MIF were markedly upregulated in OS tissues compared with the control tissues (Fig. 7A, B), whereas the expression of ODC1 showed no significant change (Fig. 7A, B). Overall, the data suggested that SNHG17 might regulate OS development by modulating CSNK2A2, MIF, and VDAC2.

## Discussion

Osteosarcoma is a bone malignancy that is most frequently found in children and adolescents [20]. Although significant advances have been made in treatment, the outcomes of this disease are still poor due to its tendency to recur and metastasize [20]. Therefore, it is urgently required to identify novel and efficient diagnostic, therapeutic, and prognostic biomarkers for this disease. In this study, by analyzing the data from GEO datasets, a large cohort of lipid metabolism-associated lncRNAs (LM-lncRNAs) were found to be closely associated with the aggressiveness of OS, and two LM-lncRNAs (SNHG17 and LINC00837) were especially enriched in OS tissues compared with the normal tissues, shown by RT-qPCR determinations of significant overexpression in both OS tissues and cell lines. These data are consistent with the findings of previous studies showing that LM-lncRNAs play critical roles in the regulation of the malignant phenotype, including tumor progression, metastasis, and drug resistance [3, 21, 22]. However, up until now, it is still unkwon whether SNHG17 and LINC00837 involve in regulating cancer progression via affecting lipid metabolism, which makes investigations on this issue especially important and necessary.

LncRNA SNHG17 belongs to the SNHG group of lncRNAs, which have been found to be significantly elevated and associated with the malignant phenotypes of tumor cells, including metastasis, proliferation, and chemical resistance [23]. Furthermore, little is known of the biological functions of LINC00837, with a few studies showing the involvement of LINC00837 in the regulation of dendritic cell functions [24], while there is no information on its role in cancer. Here, it was revealed that overexpression of both SNHG17 and LINC00837 synergistically promoted the proliferation of OS cells, whereas silencing resulted in opposite effects, which is partially supported by previous studies. Specifically, SNHG17 upregulates PAX6 to facilitate the development of oral squamous cell carcinoma [25], stimulates SOX4 to promote the epithelial-mesenchymal transition in ESCC cells [26], and activates H2AX-associated signaling pathways to promote the development of renal cell carcinoma [27]. These studies together support the hypothesis that SNHG17 is essential for sustaining the aggressiveness of multiple types of cancer. Furthermore, given that lncRNAs often exert their biological functions through serving as RNA sponges to sequester miRNA, we constructed novel lncRNA-miRNA-mRNA ce-RNA networks based on these two lncRNAs.

The tumor microenvironment is essential for sustaining the progression of multiple cancers, and recent data have suggested that lncRNAs are critical for sustaining cancer aggressiveness by regulating the tumor microenvironment [28, 29]. Hence, we hypothesized that aberrant LINC00837 and SNHG17 expression may be linked with the prognosis of OS patients and an immunosuppressive microenvironment. For evaluating the prognostic importance of LINC00837 and SNHG17, an LM-lncRNA prognostic risk model was constructed using UCRA and MCRA in the GEO training cohort. Then a prognostic nomogram integrating the RS values of the model and several clinical variables (sex, metastasis, and age) was generated and revealed that the RS efficiently predicted prognosis and was validated by the N6-methyladenosine (m6A).

-related lncRNA signature as a model in the validation cohort. Furthermore, the signature and nomogram were confirmed by ROC curves, calibration plots, Kaplan–Meier survival curves, and decision curve analysis. The AUCs of the nomogram for one-, three, and five-year survival were 0.935, 0.772, and 0.828, respectively, suggesting the effectiveness of the nomogram in predicting survival outcomes. Overall, multiple verification experiments confirmed the robustness of the risk model, indicating its potential for application to individualized risk management. It was also found that the signature-based RS values were significantly linked with metastasis, indicating that the signatures are also better predictors of OS metastasis.

GSEA was used to identify risk model-associated pathways and hallmarks in the high- and low-risk groups. KEGG analysis revealed that the LM-lncRNA risk signature was associated with both cell proliferation and metabolic processes. Aberrant lipid metabolism impairs both bone remodeling and the tumor microenvironment, leading to poor OS prognosis [30]. Previous studies have suggested that metabolic reprogramming is essential for immune cell activation, affecting the activity of the cells because of different metabolic features [31, 32]. The infiltrating immune cell levels in tumors are a critical index for assessing both prognosis and treatment impact [33]. Due to the close association of metabolic reprogramming with the tumor immune microenvironment, exploring the immune conditions in high- and low-risk groups is important. The infiltration levels of 28 different immune cells were measured in OS patients. Spearman correlation analysis indicated a marked negative correlation of RS with NK, central memory CD8-T, plasmacytoid dendritic cells, and macrophages, which are supported by the previous evidences that all the above immune cells participate in the regulation of osteosarcoma aggressiveness [34–36]. These data suggest that OS patients with reduced RSs had elevated levels of immune cell infiltration, thus it can be assumed that the RS value and immune status are associated with poor prognosis.

To elucidate how LINC00837 and SNHG17 modulate the expression of lipid metabolism-associated genes via sponging miRNAs in OS, their interactions were analyzed. This showed that while no miRNAs bind LINC00837, the SNHG17 ceRNA network indicated that SNHG17 could modulate the expression of VDAC2, ODC1, CSNK2A2, and MIF by interacting with various miRNAs. The association of these genes with OS pathogenesis and prognosis requires further research. IHC staining of tumor and adjacent healthy tissues indicated up-regulation of the VDAC2, CSNK2A2, and MIF genes in tumor tissues, although the expression levels of ODC1 did not change, possibly because of the large differences in the positive signal values between the various samples. CSNK2A2 has been shown to be a significant biomarker of prognosis in hepatocellular carcinoma and might also be linked with the pathogenesis of abdominal aortic aneurysm [37, 38]. MIF, an inflammatory cytokine linked with pathogenesis in multiple cancer types, is essentially involved in angiogenesis, immunity, and melanoma cell line metastasis [39]. Taken together, SNHG17 may, as a ceRNA, modulate the expression of lipid metabolism-associated genes, such as CSNK2A2, VDAC2, and MIF, by interacting with hsa-miR-505-3p, hsa-miR-6839-3p and hsa-miR-370-3p, and hsa-miR-451a, respectively, thereby modulating the OS initiation and progression.

In this investigation, a risk model based on two LM-lncRNA signatures was generated and validated by multiple methods, and their robustness and accuracy were verified. Furthermore, the possible modulating mechanisms of two LM-lncRNAs with prognostic importance were assessed for subsequent molecular research and OS-targeted therapy. However, this study has some limitations, due to its retrospective nature and lack of extensive experimental evidence. Therefore, an independent cohort investigation comprising immunohistochemical or PCR analyses is necessary to further verify the accuracy of the models. Despite these limitations, two LM-lncRNA signatures with a good OS prognostic value were constructed. Further research to clarify their activity in OS progression is required.

## Conclusion

In conclusion, the data indicated that SNHG17 and LINC00837 could be utilized as novel signatures for the prediction of OS prognosis. Preliminary experimental verification indicated with the lncRNAs SNHG17 and LINC00837 act as oncogenes to promote OS cell proliferation. SNHG17-microRNAs-mRNA ceRNA networks were also constructed, suggesting that downstream lipid metabolism-associated genes (CSNK2A2, MIF, and VDAC2) may be SNHG17 effector targets. This study thus identified novel signatures that could serve as biological signatures for OS diagnosis and precise OS treatment.

## Electronic supplementary material

Below is the link to the electronic supplementary material.

Supplementary Material 1

Supplementary Material 2

Supplementary Material 3

Supplementary Material 4

Supplementary Material 5

Supplementary Material 6

Supplementary Material 7

Supplementary Material 8

## Data Availability

The original contributions presented in the study are included in the article/Supplementary Material. Further inquiries can be directed to the corresponding authors. The links of the GEO datasets used in the present study were listed as follows: GSE12865: https://www.ncbi.nlm.nih.gov/geo/query/acc.cgi?acc=GSE12865. GSE16091: https://www.ncbi.nlm.nih.gov/geo/query/acc.cgi?acc=GSE16091. In addition, other original data could be obtained from the corresponding author upon reasonable request.
