# Supplementary material for: Identification of two novel lipid metabolism-related long non-coding RNAs (SNHG17 and LINC00837) as potential signatures for osteosarcoma prognosis and precise treatment
Source: BMC Med Genomics. 2023 May 25;16:115. doi: 10.1186/s12920-023-01553-4 (PMC10210430; doi:10.1186/s12920-023-01553-4)
Supplement: Supplementary file 1 — Supplementary Material 1 [file 12920_2023_1553_MOESM1_ESM.docx]

**Supplementary Table legends**

**Supplementary Table S1.** KEGG pathway analysis for the differentially expressed genes (DEGs).

**Supplementary Table S2.** The detailed expression levels of the DEGs were calculated.

**Supplementary Table S3.** The detailed expression levels of differentially expressed LncRNAs.

**Supplementary Table S4.** The prediction of LncRNAs with their potential downstream targets.
