# Supplementary figures and images for: Identification of two novel lipid metabolism-related long non-coding RNAs (SNHG17 and LINC00837) as potential signatures for osteosarcoma prognosis and precise treatment
Source: BMC Med Genomics. 2023 May 25;16:115. doi: 10.1186/s12920-023-01553-4 (PMC10210430; doi:10.1186/s12920-023-01553-4)

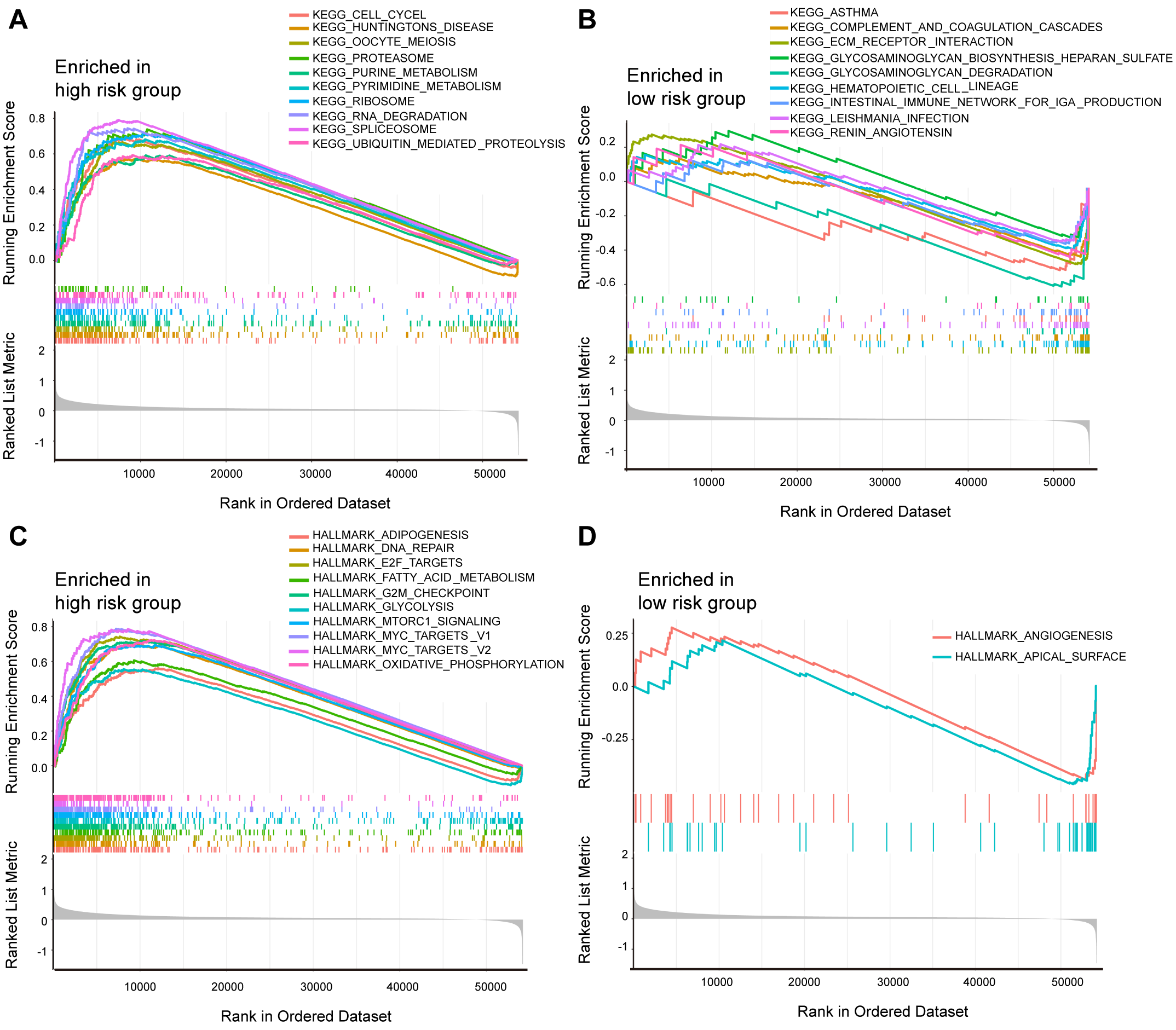

Supplement: Supplementary file 2 — Supplementary Material 2 [file 12920_2023_1553_MOESM2_ESM.tif]

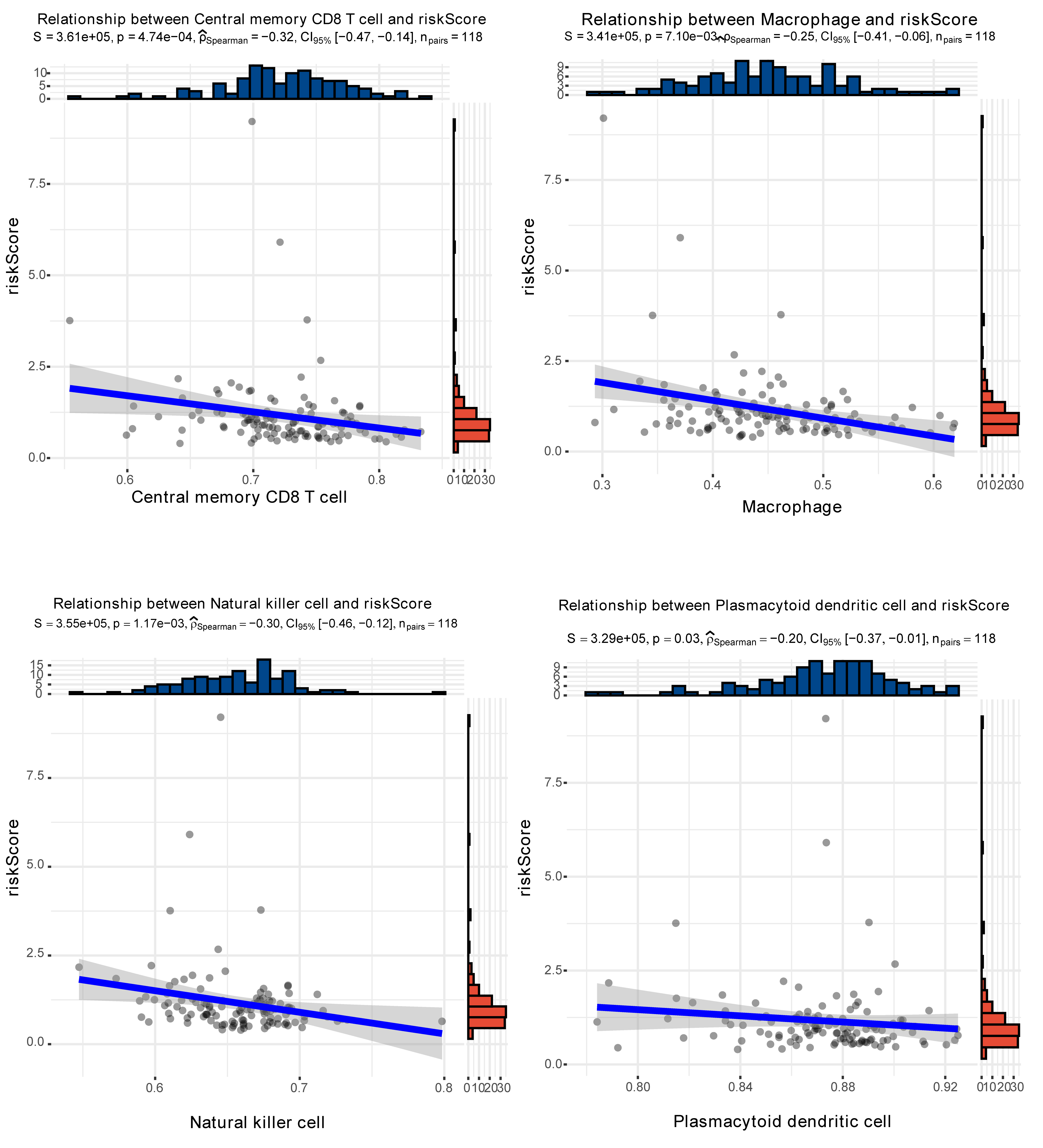

Supplement: Supplementary file 3 — Supplementary Material 3 [file 12920_2023_1553_MOESM3_ESM.tif]
