# Supplementary material for: Identification of two novel lipid metabolism-related long non-coding RNAs (SNHG17 and LINC00837) as potential signatures for osteosarcoma prognosis and precise treatment
Source: BMC Med Genomics. 2023 May 25;16:115. doi: 10.1186/s12920-023-01553-4 (PMC10210430; doi:10.1186/s12920-023-01553-4)
Supplement: Supplementary file 8 — Supplementary Material 8 [file 12920_2023_1553_MOESM8_ESM.docx]

**Supplementary Figure legends**

**Supplementary Figure S1.** GSEA plots for visualizing signaling pathways assessed by KEGG analysis and hallmarks enrichment. A: KEGG analysis in a high-risk cohort. B: KEGG analysis in the low-risk cohort. C: Hallmarks enrichment in a high-risk cohort. D: Hallmarks enrichment in the low-risk cohort.

**Supplementary Figure S2.** Relationship of risk score with immune cell infiltration in OS. ssGSEA was performed to elucidate four different immune cells’ enrichment scores in each sample. Pearson correlation of RS with each immune cell type as assessed and visualized by scatter plots.
